# Myeloid cell-specific type I interferon signaling mediates age-dependent inflammation and protection in *Bordetella pertussis* infection

**DOI:** 10.1128/iai.00306-25

**Published:** 2025-09-22

**Authors:** Amit Kumar, Alicia Bukowski, Nicholas H. Carbonetti

**Affiliations:** 1 Department of Microbiology and Immunology, University of Maryland School of Medicine 12264, Baltimore, Maryland, USA; University of Pennsylvania Perelman School of Medicine, Philadelphia, Pennsylvania, USA

**Keywords:** *Bordetella pertussis*, type I interferon, respiratory infection, innate immunity, interferon lambda, infant infection, pertussis toxin

## Abstract

Type I interferons (IFNs) play complex roles during bacterial infections. We previously found that type I IFNs were induced in *Bordetella pertussis*-infected adult mice but not in infant mice, a potentially relevant clinical dichotomy, since pertussis can be fatal in human infants. We investigated the role of type I IFNs and their cross-regulation with type III IFNs (IFN-λ) in *B. pertussis* infection across developmental stages. In contrast to global IFNAR1 knockout adult mice, in which lung inflammation was equivalent to that in wild-type mice, myeloid cell-specific deficiency of the type I IFN receptor protein IFNAR1 (LysM^Cre^IFNAR1^fl/fl^) resulted in significantly reduced lung inflammation and pro-inflammatory cytokine production, despite elevated pulmonary IFN-λ levels. Mechanistically, we found that, in contrast to WT macrophages, IFNAR1-deficient macrophages produced IFN-λ in response to *B. pertussis* or pertussis toxin, a process dependent on the G protein-coupled receptor lysophosphatidic acid receptor 1 (LPAR1). IFNAR1 deficiency did not affect type I IFN expression or killing capacity by macrophages and neutrophils. In striking contrast to WT infant mice, which developed resistance to lethal *B. pertussis* infection by postnatal day 10 (P10), LysM^Cre^IFNAR1^fl/fl^ infant mice remained highly susceptible to lethal infection through P21, exhibiting increased lung bacterial burden and inflammation, as well as increased bacterial dissemination compared to WT infant mice. These findings reveal a critical age- and cell-specific interplay between type I and III IFNs during *B. pertussis* infection and highlight a novel LPAR1-dependent pathway for IFN-λ induction in the absence of type I IFN signaling.

## INTRODUCTION

Pertussis, a serious respiratory bacterial infection, has re-emerged at high levels after a lull in the number of cases during the COVID-19 pandemic. In 2024, the U.S.A. reported the highest number of cases since 2012, and 10 infants died from pertussis (https://www.cdc.gov/pertussis/php/surveillance/index.html). However, we still have a relatively poor understanding of the pathogenesis of pertussis disease, and no consistent effective therapies exist for treatment of individuals suffering from this debilitating infection.

We have shown that age-dependent outcomes of experimental *B. pertussis* infection in mice parallel aspects of human pertussis, such as pulmonary hypertension and leukocytosis seen in infants but not adults (1–3). Infant mice suffer a lethal disseminating infection, while adult mice restrict infection to the respiratory tract, mount inflammatory responses, and recover (2, 4). Recently, we have investigated the role of interferons (IFNs) in this host–pathogen interaction in mouse models. We found that *B. pertussis-*infected adult mice upregulate expression of type I (IFN- α/β), type II (IFN-γ), and type III (IFN-λ) IFNs in the lungs, whereas infected infant mice inoculated at postnatal day 7 (P7) do not upregulate these IFNs (4–6). IFN-γ plays a protective role against *B. pertussis* infection in adult and infant mice (4, 7, 8), but the roles of the other IFNs during *B. pertussis* infection are less well understood.

Type I IFNs and type III IFNs (IFN-λ) share several common properties and induce an overlapping set of IFN-stimulated genes (ISGs) (9). A key difference between these IFNs is that the type I IFN receptor (IFNAR) is expressed on almost all mammalian cells, while the IFN-λ receptor (IFNLR) is expressed on a limited set of cell types, including epithelial cells, neutrophils, and some other immune cells (9). IFN-λ has diverse effects on experimental bacterial infection in mice, being protective in some and deleterious in others (10–12). We found that IFN-λ has significant inhibitory effects on several antibacterial activities of neutrophils in *B. pertussis-*infected adult mice, promoting bacterial growth and lung inflammation (13).

Type I IFNs, of which IFN-α and IFN-β are the best studied, bind their unique dimeric receptor (IFNAR1 and IFNAR2) and initiate signaling through a pathway involving JAK1, TYK2, STAT1, STAT2, and IRF9 to induce many IFN-stimulated genes (ISGs) (14). Type I IFNs are key cytokines in immune responses and antiviral defense (15), but they also contribute to inflammation and pathogenesis in a variety of disease models (16–18). Like IFN-λ, type I IFNs have diverse effects on bacterial infections in experimental models (19). The varied activities of type I IFNs combined with the specific context of each host–pathogen interaction likely determine this dichotomy of effects.

Our previous study indicated that type I IFNs exacerbate lung inflammation in *B. pertussis-*infected adult mice (5). However, this conclusion was complicated by the observation that infected IFNAR1-deficient mice upregulated expression of lung IFN-λ to significantly greater levels than infected wild-type mice and that this higher level of IFN-λ promoted lung inflammation (5). In addition, we saw age-dependent effects of type I IFNs in *B. pertussis* infection outcomes. In *B. pertussis-*infected IFNAR1-SA knock-in mice, in which the IFNAR receptor cannot be internalized/degraded and signals constitutively at the surface (20), adult mice had exacerbated levels of lung inflammation, but infant mice were relatively protected from the lethal effects of infection (5). However, the target cells and mechanisms mediating these type I IFN effects are unclear.

Since myeloid cells play important roles in protection against *B. pertussis* infection (21–23) and type I IFNs have diverse effects on myeloid cells (24–27), we investigated the role of type I IFN signaling specifically in myeloid cells by constructing a mouse strain with myeloid-specific knockout (KO) of IFNAR1. Studying outcomes of *B. pertussis* infection in these mice revealed age-dependent effects of myeloid-specific type I IFN signaling, including effects on lung IFN-λ expression as observed in whole-body IFNAR1 KO mice.

## MATERIALS AND METHODS

### Bacterial strains

In these studies, we used a strain of *B. pertussis* that is a streptomycin-resistant derivative of Tohama I^28^. *B. pertussis* strain ΔPT was described previously (28). We cultured *B. pertussis* on Bordet–Gengou agar, which was supplemented with 10% defibrinated sheep blood (Lampire Biological Laboratories) and 200 µg/mL streptomycin. The cultures were incubated at 37°C for 48 hours.

### Mouse infections and treatment

All mouse strains were maintained on a C57BL/6 genetic background. LysMCre, IFNAR1^fl/fl^, and IFNAR1 knockout (IFNAR1^-/-^) mice were obtained from the Jackson Laboratory. All animal experiments were conducted in accordance with protocols approved by the Institutional Animal Care and Use Committee at the University of Maryland, Baltimore. *B. pertussis* was cultured for 48 hours on Bordet–Gengou agar, and bacterial suspensions were prepared in phosphate-buffered saline (PBS). Adult mice were infected intranasally with 2 × 10^6^ colony-forming units (CFU) in a 50 µL volume. For infant mice, bacteria were administered by aerosol, as described previously (2). Following infection, the lungs and other organs were harvested to assess bacterial burden, gene and protein expression, immune cell infiltration, and inflammatory histopathology. For survival studies, mice over 21 dpi were observed when the experiment was terminated. Generally, groups of adult mice consisted of at least four animals of both sexes, and groups of infant mice were litters of five to nine animals of both sexes.

### Cell culture

Bone marrow cells were harvested from the femurs and tibias of 6- to 8-week-old C57BL/6 J mice and differentiated into bone marrow-derived macrophages (BMDMs) by culturing in RPMI 1640 medium (Gibco) supplemented with 10% fetal bovine serum (FBS; Gibco), 100 U/mL penicillin, 100 µg/mL streptomycin, 2 mM L-glutamine (Gibco), and 50 ng/mL recombinant mouse macrophage colony-stimulating factor (M-CSF; PeproTech). Cells were incubated at 37°C in a humidified 5% CO₂ incubator for seven days. Peritoneal macrophages were isolated from 6- to 8-week-old C57BL/6 J mice. Mice were injected intraperitoneally with 0.5 mL of 4% thioglycolate, and peritoneal exudate cells were collected by lavage three days post-injection. Cells were washed twice with PBS and plated in 6-well plates. After a 4-hour incubation at 37°C, nonadherent cells were removed by washing with PBS, and the adherent macrophages were used for downstream experiments. Neutrophils were isolated from bone marrow using a 62.5% Percoll gradient (GE Healthcare) in HBSS. Cell purity was confirmed by flow cytometry and was consistently ≥92%. For *in vitro* stimulation, neutrophils or macrophages (2 × 10⁶ cells/mL) were pretreated with 100 ng/mL recombinant mouse IFN-λ2 or IFN-β (R&D Systems) for 30 minutes at 37°C. Cells were then stimulated with either wild-type *B. pertussis*, *B. pertussis* ΔPT (multiplicity of infection [MOI] 10), 200 ng/mL pertussis toxin [PT], or inactive mutant PT [PT*] for 30 minutes. Prior to stimulation, bacteria were centrifuged onto cells at 600 × *g* for 5 minutes. After incubation, the medium was removed and replaced with HBSS containing 20 µg/mL polymyxin B to eliminate extracellular bacteria.

### Isolation and flow cytometry analysis of lung cells

For intracellular cytokine staining of IFN-γ, 250 µg brefeldin A (BioLegend) was injected intraperitoneally into mice 6 hours prior to lung harvest for analysis. Three lung lobes (superior, middle, and inferior) were isolated and sliced and were digested with collagenase-D (1 mg/mL; Roche) and DNase I (10 mg/mL; Sigma-Aldrich) for 1 hour at 37°C with agitation. Ammonium-chloride-potassium buffer was used to lyse red blood cells. The single-cell suspension was washed with PBS, and a total of 2 × 10^6^ cells were stained with LIVE/DEAD™ Fixable Aqua Dead Cell Stain Kit (Invitrogen), followed by incubation with Fc block (CD16/CD32) (BioLegend) to block IgG Fc receptors. Cells were then stained with antibodies at 4°C for 30 minutes. Flow cytometry antibodies used were BV650-CD45 (Clone 30-F11, BioLegend), PerCP-Ly6G (clone 1A8, BioLegend), and BV711- CD11b (clone M1/70, BioLegend). After staining, cells were fixed with 2% PFA for 10 minutes at room temperature, washed, and resuspended in 350 µL of FACS buffer and acquired on an Aurora Spectral Flow Cytometer (Cytek). Analysis was performed using FlowJo 10.10 (BD).

### Immunoblotting

Cell lysates of macrophages were obtained by lysis in RIPA buffer. Supernatants were collected after centrifugation at 16,000 × *g* for 15 minutes, and protein concentration was measured using a BCA protein assay kit (Pierce). Twenty micrograms of protein were loaded on 10–12% sodium dodecyl sulfate (SDS) polyacrylamide gels and transferred to PVDF membranes. After blocking with 5% BSA in TBST, membranes were probed with primary antibodies to pSTAT1 (BD Biosciences), total STAT1, and beta-actin (CST), followed by incubation with secondary antibodies. Western blots were developed using a Gel Doc imaging system (Image Quant LS 500; Biorad).

### RNA processing and quantitative real-time polymerase chain reaction

Total RNA was extracted using TRIzol reagent (Invitrogen) and reverse transcribed into cDNA using the Reverse Transcription Kit (Promega), according to the manufacturer’s instructions. Quantitative real-time PCR was performed using the Maxima SYBR Green/ROX qPCR Master Mix (Thermo Fisher Scientific) on an Applied Biosystems 7500 Fast Real-Time PCR System. Expression levels were normalized to the housekeeping gene hypoxanthine phosphoribosyltransferase (Hprt). Primer sequences are listed in Table S1. Relative gene expression was calculated using the 2–ΔΔCT method, with PBS-treated (“sham”-infected) animals serving as the reference control

### Histopathology

Lungs were perfused with phosphate-buffered saline (PBS) and fixed in 10% (wt/vol) buffered formalin. Hematoxylin and eosin (H&E) staining was performed by the Pathology and Histology Laboratory at the University of Maryland School of Medicine. Histopathological evaluation was conducted in a blinded manner by two independent investigators. Each lung section was scored in three categories—tissue consolidation, severity of bronchovascular bundle inflammation, and the percentage of bronchovascular bundles affected—using a 0–3 scale per category, resulting in a cumulative histopathology score ranging from 0 to 9.

### Enzyme-linked immunosorbent assay

After harvest, tissues were flash-frozen in an isopropanol/dry ice bath. The tissue was homogenized in 1 to 2 mL PBS with a protease inhibitor to prevent protein degradation. According to the manufacturer’s protocol, an enzyme-linked immunosorbent assay (ELISA) was performed on dilutions of the tissue samples. To evaluate cytokine production in the supernatants, 2 × 10^6^ neutrophils or macrophages were plated in 6-well plates and stimulated with *B. pertussis*. The ELISA evaluated the production of IFN-λ (R and D Systems).

### Bacterial killing assay

Neutrophils and macrophages were infected with *B. pertussis* at a multiplicity of infection (MOI) of 10. Bacteria were centrifuged onto cells at 600 × *g* for 5 minutes and incubated at 37°C for 30 minutes. After incubation, cells were washed and cultured for an additional 4 hours (for neutrophils) and 24 hours in HBSS medium containing 20 µg/mL polymyxin B to eliminate adherent extracellular bacteria. Following this, cells were washed thoroughly, and neutrophils were lysed in sterile water. Lysates were plated on Bordet–Gengou agar for the enumeration of intracellular colony-forming units (CFUs).

### Statistical analysis

Data analysis and graph generation were performed using GraphPad Prism version 10.0 (GraphPad Software). Results are presented as mean ± standard error (SE). Statistical significance between two groups was assessed using an unpaired Student’s *t*-test. For comparisons among multiple groups, one-way analysis of variance followed by Tukey’s multiple comparisons test was used. A *P* value of < 0.05 was considered statistically significant.

## RESULTS

### IFNAR1 signaling in myeloid cells exacerbates lung inflammatory pathology in *B. pertussis*-infected adult mice

**Fig 1:**
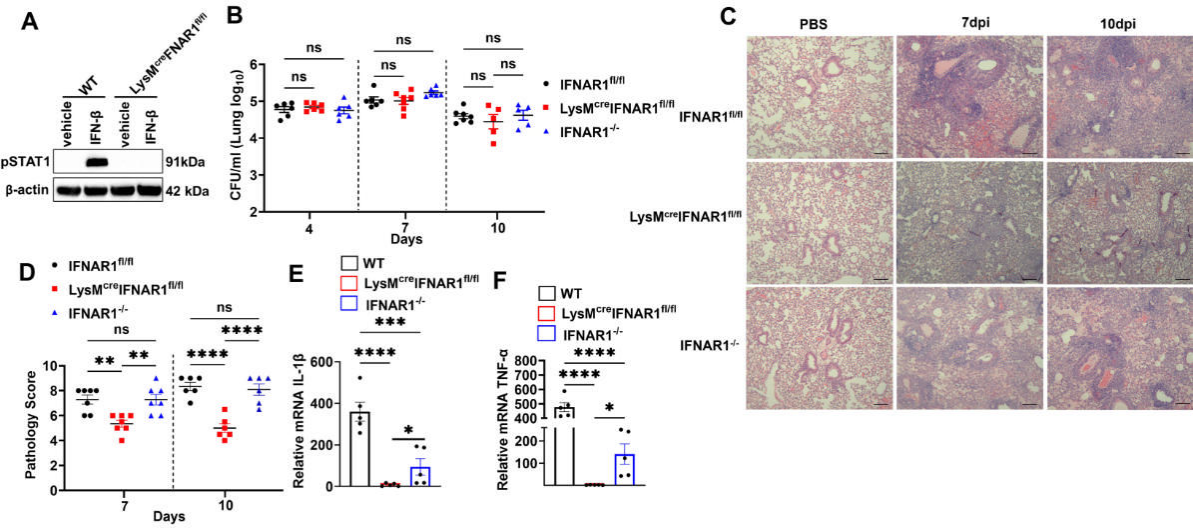
IFNAR1 signaling in macrophages exacerbates lung inflammation in *B. pertussis***-**infected adult mice (**A**) STAT1 phosphorylation in IFNAR1^fl/fl^ and LysM^cre^IFNAR1^fl/fl^ BMDMs incubated with IFN-β (100 ng/mL) for 60 minutes. (**B–E**) IFNAR1^fl/fl^, LysM^cre^IFNAR1^fl/fl^, and IFNAR1^-/-^ mice (*n* ≥ 5 per group) were euthanized at 4, 7, and 10 dpi with *B. pertussis*, or PBS sham inoculum and lungs were dissected for assessment of outcomes. (**B**) Bacterial burdens. (**C**) Representative H&E-stained lung sections (210 µm). (**D**) Inflammatory pathology scores were assessed from lung histology sections. Relative mRNA levels of (**E**) IL-1β and (**F**) TNF-α were assessed by quantitative RT-PCR (infected versus sham inoculated). *P < 0.05, **P < 0.01, ***P < 0.001, ****P < 0.0001, ns, not significant, by one-way ANOVA.

Type I interferons (IFNs) are widely recognized for their pro-inflammatory roles in various infection and disease models (15). In our previous studies, we demonstrated that type I IFNs exacerbate lung inflammation during *B. pertussis* infection in adult mice (5). To investigate cell types that may be involved in this effect, we constructed myeloid cell-specific IFNAR1 knockout mice (LysM^cre^IFNAR1^fl/fl^) and examined the ability of IFN-β to induce STAT1 phosphorylation in bone marrow-derived macrophages (BMDMs) from these and control (IFNAR1^fl/fl^) mice. In control macrophages, IFN-β induced robust STAT1 phosphorylation, whereas this response was abrogated in macrophages from LysM^cre^IFNAR1^fl/fl^ mice (Fig. 1A). This confirmed the loss of functional IFNAR1 in macrophages from LysM^cre^IFNAR1^fl/fl^ mice. To assess the role of myeloid-specific IFNAR1 signaling in *B. pertussis* pathogenesis, we examined bacterial loads and lung inflammation in control (IFNAR1^fl/fl^), LysM^cre^IFNAR1^fl/fl^, and IFNAR1^−/−^ mice. All three groups displayed similar lung bacterial loads at 4, 7, and 10 days post-inoculation (dpi) (Fig. 1B). However, while IFNAR1^−/−^ mice showed similar levels of lung inflammation to control mice, LysM^cre^IFNAR1^fl/fl^ mice exhibited significantly reduced lung inflammation and lower inflammatory pathology scores at 7 and 10 dpi (Fig. 1C and D). We then evaluated the production of pro-inflammatory cytokines IL-1β and TNF-α in the lungs of these infected mice. Notably, LysM^cre^IFNAR1^fl/fl^ mice showed a significant reduction in cytokine production compared to both IFNAR1^−/−^ and control mice, in concordance with their reduced lung inflammation (Fig. 1E and F). In contrast, IFNAR1^−/−^ mice had lower levels of IL-1β and TNF-α compared to controls, yet the lung inflammation did not correlate with these reductions (Fig. 1E and F). These results suggest that while type I IFNs contribute to pro-inflammatory cytokine production and lung inflammation, myeloid cell-specific IFNAR1 deletion significantly reduces inflammation without affecting bacterial clearance. This highlights the critical role of myeloid cell IFNAR1 signaling in promoting lung inflammation during *B. pertussis* infection.

### Loss of IFNAR1 signaling in macrophages increases type III IFN secretion

**Fig 2:**
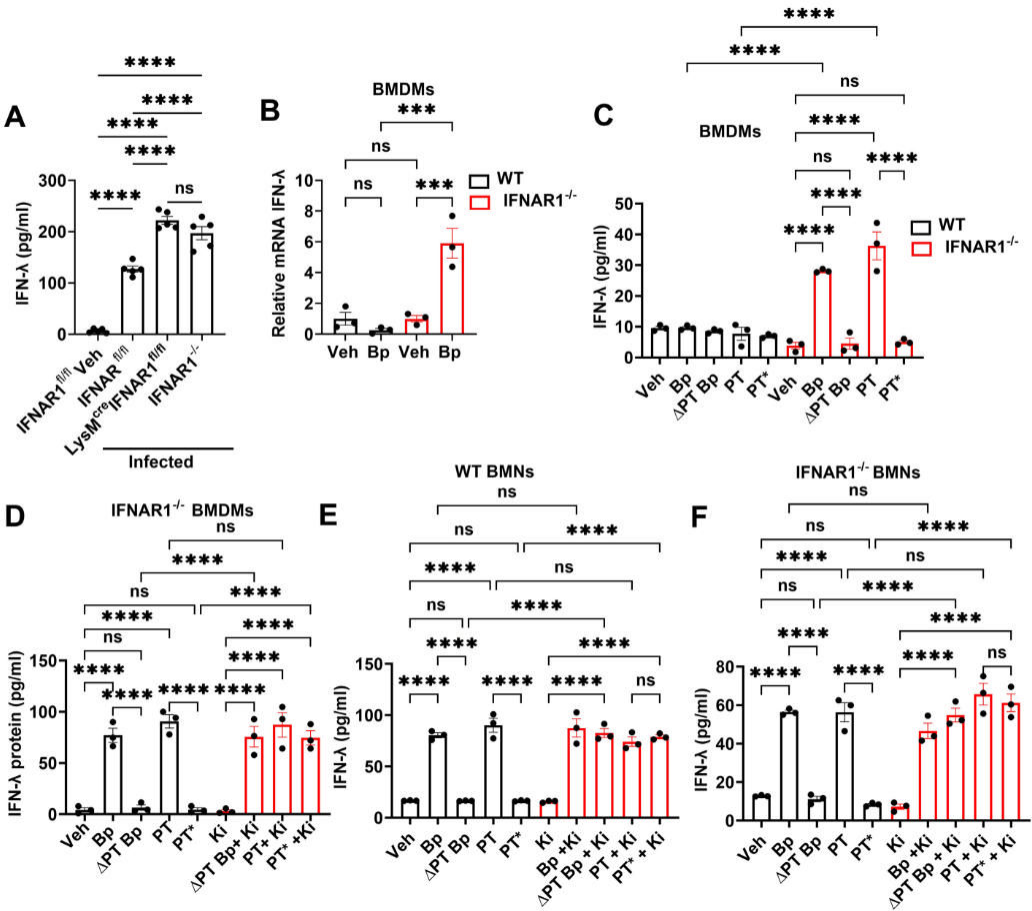
Pertussis toxin-mediated inhibition of the GPCR lysophosphatidic acid receptor 1 induces type III interferon production in IFNAR1-deficient macrophages and neutrophils. (**A**) Adult mice (*n* > 4) were euthanized at 7 dpi with *B. pertussis*, or PBS sham inoculum and lungs were dissected for assessment of IFN-λ protein levels by ELISA. (**B–F**) Type III IFN responses of wild-type (WT) and IFNAR1^⁻/⁻^ macrophages and neutrophils following stimulation with *B. pertussis* (Bp, MOI 10), PT-deficient *B. pertussis* (ΔPT Bp, MOI 10), pertussis toxin (PT, 200 ng/L), or an enzymatically inactive PT (PT*, 200 ng/mL), with or without the LPAR1 inhibitor Ki16425 (10 µM). Samples were collected at 4 hours (neutrophils) or 8 hours (macrophages) post-stimulation. (**B**) IFN-λ mRNA in BMDMs, and IFN-λ protein levels in (**C**) BMDMs, (**D**) IFNAR1^-/-^ BMDMs, (**E**) WT bone marrow neutrophils (BMNs), and (**F**) IFNAR1^-/-^ BMNs. ***P < 0.001, ****P < 0.0001, ns, not significant, by one-way ANOVA.

We previously reported that *B. pertussis*-infected IFNAR1^⁻/⁻^ mice exhibit lung inflammation compared to wild-type (WT) mice but display significantly higher IFN-λ gene expression in lung tissue. Neutralization of IFN-λ using blocking antibodies reduced lung pathology in IFNAR1^⁻/⁻^ mice (5). In contrast, *B. pertussis*-infected LysM^Cre^IFNAR1^fl/fl^ mice exhibited markedly reduced lung inflammation compared to both WT and IFNAR1^⁻/⁻^ mice (Fig. 1C and D). To determine whether this decreased inflammation was due to lower IFN-λ expression, we assessed IFN-λ production in these infected mice. In both LysM^Cre^IFNAR1^fl/fl^ and IFNAR1^⁻/⁻^ mice, IFN-λ protein levels were significantly elevated relative to control IFNAR1^fl/fl^ mice (Fig. 2A), whereas type I IFNs (IFN-β and pan-IFN-α) remained unchanged (Fig. S1A and B). Therefore, the lower level of lung inflammation in LysM^Cre^IFNAR1^fl/fl^ mice was not due to lower IFN-λ protein levels.

Given our prior findings that neutrophils secrete IFN-λ in response to PT (13), we examined IFN-λ induction in BMDMs and peritoneal macrophages (PMs) following infection *in vitro*. IFN-λ mRNA was upregulated at 8 hours post-infection in IFNAR1^⁻/⁻^ BMDMs (Fig. 2B) and PMs (Fig. S2) but not in WT macrophages. We further identified PT as a key factor regulating IFN-λ production in IFNAR1^⁻/⁻^ macrophages. IFNAR1^⁻/⁻^ BMDMs infected with ΔPT *B. pertussis* (lacking PT) failed to induce IFN-λ (Fig. 2C). Treatment of IFNAR1^⁻/⁻^ BMDMs with purified PT (200 ng/mL, 8 hours) led to robust induction of IFN-λ expression, whereas treatment with an enzymatically inactive version of PT (29) did not induce IFN-λ (Fig. 2C), indicating that the ADP-ribosyltransferase activity of PT is required for IFN-λ induction. Neither bacteria nor PT induced IFN-λ in WT BMDMs (Fig. 2C).

We hypothesized that PT promotes IFN-λ expression in myeloid cells by inhibiting signaling through lysophosphatidic acid receptor 1 (LPA1). This was based on previous reports that LPA1 is expressed on myeloid cells (30), is a Gi protein-coupled receptor (GPCR) (29) targeted by PT inhibition, and that LPA1 signaling negatively regulates IFN-λ responses in virus-infected macrophages (31). To test this, IFNAR1^⁻/⁻^ BMDMs were exposed to ΔPT bacteria or PT* in the presence of the LPA1 inhibitor Ki1642532 (32). LPA1 inhibition restored IFN-λ production to levels comparable to BMDMs infected with WT *B. pertussis* or treated with PT (Fig. 2D). These findings support a model in which PT inhibits LPA1 signaling-mediated suppression of IFN-λ expression, thus resulting in increased IFN-λ production.

Consistent with these findings, IFN-λ secretion was also observed in both WT and IFNAR1^⁻/⁻^ neutrophils, dependent on inhibition of LPA1 signaling by PT following *B. pertussis* infection (Fig. 2E and F). No significant difference in IFN-λ levels was detected between the WT and IFNAR1^-/-^ neutrophils, indicating that the presence of the type I IFN receptor does not influence PT-induced IFN-λ production in neutrophils.

Together, these results demonstrate that IFNAR1 signaling inhibits IFN-λ production in macrophages, and that PT induces IFN-λ expression by targeting and inactivating Gi-coupled LPA1 signaling pathways in IFNAR1^-/-^ macrophages, as well as WT and IFNAR1^-/-^ neutrophils. This identifies a novel regulatory pathway, whereby PT modulates LPA1 signaling to promote type III IFN responses, particularly in the absence of IFNAR1-mediated signaling as observed in the lungs of IFNAR1^-/-^ and LysM^Cre^IFNAR1^fl/fl^ mice.

### Regulation of type I IFN production in IFNAR1^-/-^ macrophages

**Fig 3:**
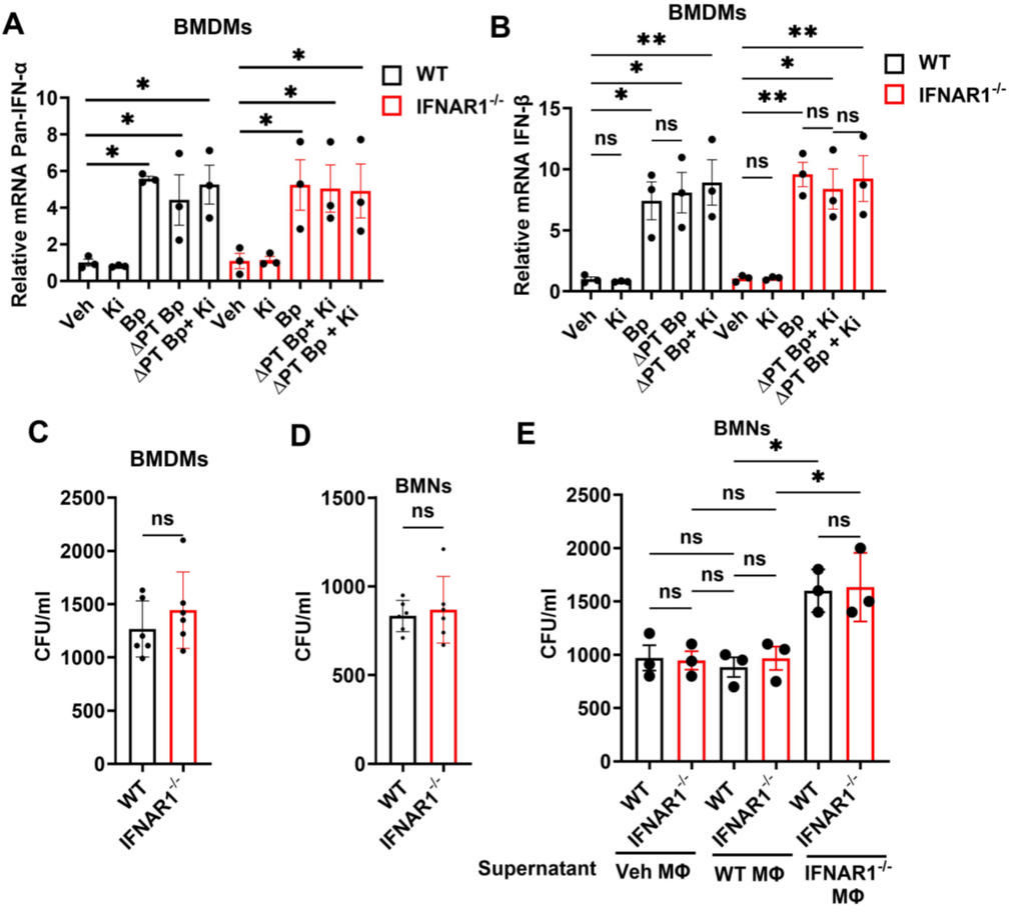
Regulation of type I IFN production in IFNAR1^-/-^ macrophages. (**A–B**) Samples were collected at 8 hours post-stimulation of wild-type (WT) and IFNAR1^-/-^ BMDMs by Bp (MOI 10) or ΔPT Bp (MOI 10), with or without the LPAR1 inhibitor Ki16425 (10 µM), and assessed for relative gene expression (vs. vehicle control) of (**A**) Pan-IFN-α and (**B**) IFN-β. (**C–D**) Bacterial CFU/mL recovered from WT or IFNAR1^−/−^. (**C**) BMDMs incubated with *B. pertussis* MOI 10 for 8 hours or (**D**) BMNs incubated with *B. pertussis* MOI 10 for 4 hours. (**E**) Bacterial CFU/mL recovered from WT or IFNAR1^−/−^ BMNs incubated with *B. pertussis* MOI 10 for 30 minutes and then further incubated with supernatants from IFNAR1^-/-^ BMDMs incubated with vehicle (Veh MΦ), WT BMDMs incubated with Bp (MOI 10 for 8 hours), or IFNAR1^−/−^ BMDMs incubated with Bp (MOI 10 for 8 hours).*P < 0.05, **P < 0.01, ns, not significant, by one-way ANOVA.

Since type I IFN receptor deficiency influenced IFN-λ production by macrophages, we investigated whether the same was true of type I IFN production. In contrast to IFN-λ production, both WT and IFNAR1⁻/⁻ BMDMs exposed to *B. pertussis* induced type I IFN (IFNα and IFN-β) expression, and this induction was independent of PT (Fig. 3A and B). These data demonstrate that signaling through IFNAR1 does not affect type I IFN induction in BMDMs, in contrast to its suppression of IFN-λ production. We next examined the effect of type I IFN signaling on the intracellular survival of *B. pertussis* in macrophages (Fig. 3C) and neutrophils (Fig. 3D). Cells were infected with *B. pertussis*, washed after 30 minutes to remove extracellular bacteria, and then cultured for an additional 8 hours (macrophages) or 4 hours (neutrophils) before being lysed to determine intracellular bacterial load by CFU quantification. Consistent with lung CFU data from infected LysM^Cre^IFNAR1^fl/fl^ and IFNAR1^⁻/⁻^ mice, there was no significant difference in bacterial killing between WT and IFNAR1^⁻/⁻^ macrophages or neutrophils (Fig. 3C and D). Previously, we showed that IFN-λ impairs neutrophil bactericidal function by inhibiting ROS production, MMP9, and MPO activity—factors critical for bacterial clearance (13). Given that IFNAR1^⁻/⁻^ macrophages secrete IFN-λ, we assessed neutrophil killing capacity in the presence of macrophage supernatants. Neutrophils were infected with *B. pertussis* and, after 30 minutes, washed and cultured for 4 hours with supernatants from WT or IFNAR1⁻/⁻ macrophages that had been either vehicle-treated or infected with *B. pertussis* (MOI 10, 8 hours). No significant difference in bacterial killing was observed when neutrophils (WT or IFNAR1^⁻/⁻^) were cultured with vehicle or with infected WT macrophage supernatants (Fig. 3E). However, a significant reduction in bacterial killing (increase in CFU) was observed in both WT and IFNAR1^⁻/⁻^ neutrophils cultured with infected IFNAR1^⁻/⁻^ macrophage supernatants. In contrast to the *in vivo* lung CFU data from WT, LysM^Cre^IFNAR1^fl/fl^, and IFNAR1^⁻/⁻^ mice, this macrophage–neutrophil co-culture experiment revealed an inhibitory effect on neutrophil function mediated by IFNAR1^⁻/⁻^ macrophage-derived factors, likely IFN-λ.

### Loss of IFNAR signaling in myeloid cells increases susceptibility to lethal *B. pertussis* infection in infant mice

**Fig 4:**
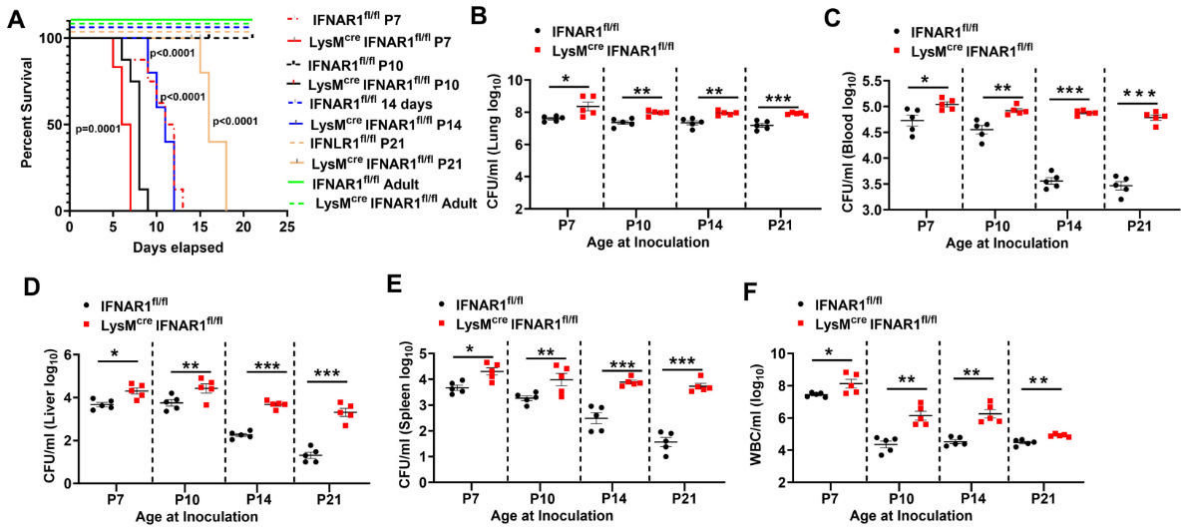
Loss of type I IFN signaling in myeloid cells increases susceptibility to lethal *B. pertussis* infection in infant mice. Infant control IFNAR1^fl/fl^ and LysM^cre^IFNAR1^fl/fl^ mice (*n* ≥ 4 per group) at the indicated postnatal (**P**) ages or 6- to 8-week-old (adult) mice were inoculated with *B. pertussis*. At 6 dpi (**P7**) and 7 dpi (P10, P14, P21, adult), mice were euthanized and organs harvested, while separate cohorts were monitored for survival until 21 dpi. (**A**) Kaplan–Meier survival curves of mice infected with *B. pertussis* at the indicated postnatal ages. (**B–E**) Bacterial burden quantified as CFUs from (**B**) lungs, (**C**) blood, (**D**) liver, and (**E**) spleen at 7 dpi. (**F**) White blood cell (WBC) counts in blood at 7 dpi. *P < 0.05, **P < 0.01, ***P < 0.001 by one-way ANOVA. Survival significance determined by log-rank (Mantel–Cox) test.

In human infants, *B. pertussis* infection can lead to severe disease characterized by marked leukocytosis, pulmonary hypertension, and, in some cases, death (3). We previously characterized a postnatal day 7 (P7) infant mouse model of *B. pertussis* infection and found that WT infant mice were highly susceptible to lethal infection at P7, whereas WT mice inoculated at P10 or later were resistant to lethal disease (2, 4). To investigate age-associated resistance to *B. pertussis* in mice lacking type I IFN signaling in myeloid cells, we infected control IFNAR1^fl/fl^ and LysM^Cre^IFNAR1^fl/fl^ mice at increasing postnatal ages (P7, P10, P14, and P21), as well as in adulthood (6–8 weeks). We assessed bacterial burdens, leukocytosis, and survival. Notably, LysM^Cre^IFNAR1^fl/fl^ mice were highly susceptible to lethal infection at all ages (P7–P21) other than adulthood, whereas control mice of all ages other than P7 survived infection (Fig. 4A). All P7-P21 LysM^Cre^IFNAR1^fl/fl^ mice died from infection, although time to death increased with increasing age, and P7 LysM^Cre^IFNAR1^fl/fl^ mice died significantly earlier than P7 WT mice (Fig. 4A). Across all P7–P21 ages, LysM^Cre^IFNAR1^fl/fl^ mice exhibited significantly higher lung bacterial burdens (Fig. 4B); increased bacterial dissemination to the blood (Fig. 4C), liver (Fig. 4D), and spleen (Fig. 4E); and exacerbated leukocytosis (Fig. 4F), compared to control mice. These findings indicate that type I IFN signaling in myeloid cells plays a critical role in bacterial clearance during infant *B. pertussis* infection and that its absence renders mice susceptible to lethal infection at ages at which WT mice are resistant. Interestingly, in infected adult mice, LysM^Cre^IFNAR1^fl/fl^ animals did not show significant differences in lung bacterial burdens compared to controls (Fig. 1B), whereas infant mice demonstrated increased bacterial load and dissemination. This suggests that the impact of myeloid-specific type I IFN signaling on host defense against *B. pertussis* is developmentally regulated and more critical at younger ages.

### Lack of IFNAR1 signaling on myeloid cells exacerbates lung inflammation in *B. pertussis*-infected infant mice

**Fig 5:**
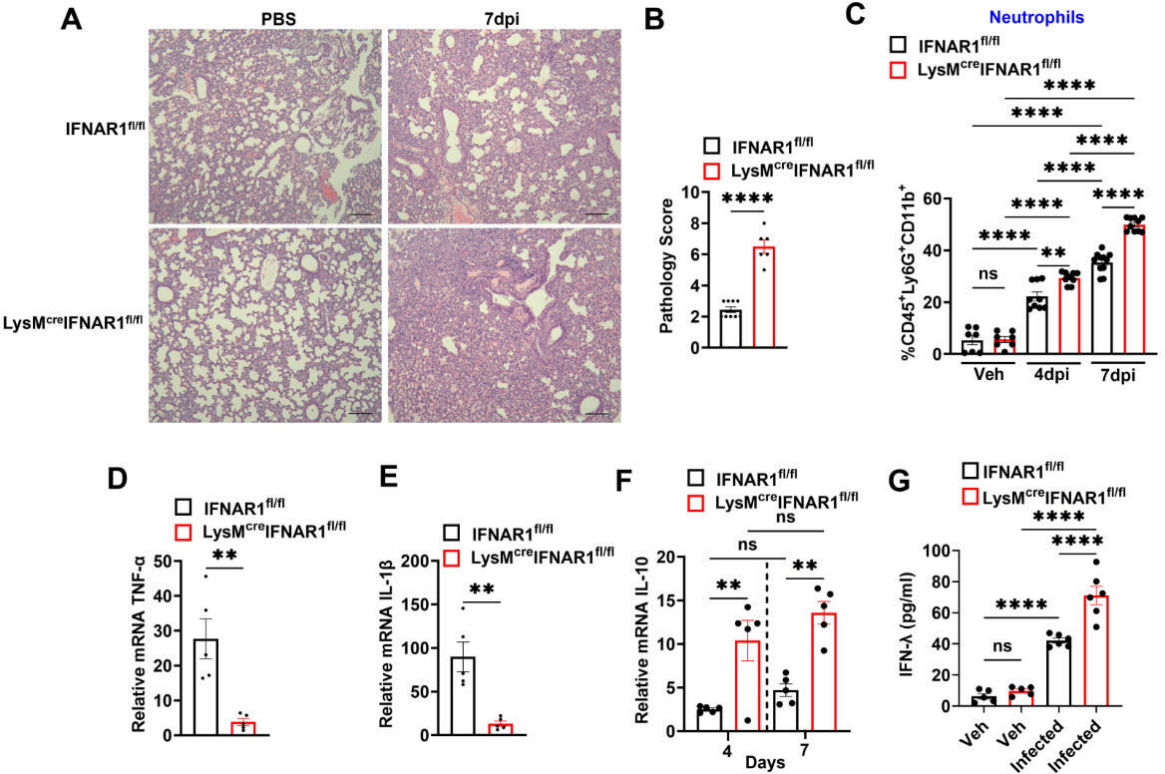
Lack of IFNAR1 signaling exacerbates lung inflammation in *B. pertussis*-infected infant mice. Infant IFNAR1^fl/fl^ and LysM^cre^ IFNAR1^fl/fl^ mice (*n* ≥ 4 per group) were inoculated at P10 with *B. pertussis* or PBS (sham control) and euthanized at 7 dpi. Lungs were harvested for analysis of inflammation and immune responses. (**A**) Representative H&E-stained lung sections showing inflammatory pathology in uninfected and infected mice (scale bar: 210 µm). (**B**) Quantification of inflammatory pathology scores. (**C**) Infant IFNAR1^fl/fl^ and LysM^cre^IFNAR1^fl/fl^ mice (*n* ≥ 4 per group) were inoculated at P10 with *B. pertussis* or PBS (sham control), euthanized at 4 and 7 dpi, and lung single-cell suspensions were analyzed by flow cytometry for neutrophil influx. (**D**–**F**) Fold induction (infected vs. sham) of lung pro- and anti-inflammatory cytokine gene expression at 7 dpi: (**D**) TNF-α, (**E**) IL-1β, and (**F**) IL-10. (**G**) IFN-λ protein levels in lung homogenates at 7 dpi. **P < 0.01, ****P < 0.0001, ns, not significant, by one-way ANOVA.

Classical pertussis is characterized by a severe paroxysmal cough in children and adults, but in infants, this cough is often weak or absent (3). In experimental animal models of pertussis, lung inflammation is commonly used as a surrogate marker of disease severity (33). We previously described pulmonary inflammation and cytokine responses in *B. pertussis*-infected adult and infant mice. Unlike the robust lung inflammation in adult mice, infant mice displayed significantly lower levels of cellular infiltration and inflammatory cell cuffing around bronchovascular bundles at 7 dpi (2). In contrast, LysM^Cre^IFNAR1^fl/fl^ infant mice inoculated at P10 exhibited heightened lung inflammation at 7 dpi (Fig. 5A and B).

Surprisingly, this was accompanied by a greater influx of neutrophils into the lung at 4 and 7 dpi (Fig. 5C), as assessed using the gating strategy shown in Fig. S3, despite no significant changes in expression of the neutrophil-attracting chemokines CXCL1 and CXCL2 (Fig. S4). Interestingly, loss of type I IFN signaling in myeloid cells decreased the production of pro-inflammatory cytokines TNF-α and IL-1β at 7 dpi (Fig. 5D and E). IL-10 is an immunosuppressive cytokine that limits inflammation to prevent excessive lung pathology during *B. pertussis* infection, although this also hinders bacterial clearance (34). We observed increased expression of lung IL-10 in LysM^Cre^IFNAR1^fl/fl^ mice compared to control mice at 4 and 7 dpi (Fig. 5F), which may contribute to the lower levels of pro-inflammatory cytokines, even though overall inflammation was higher in these mice (Fig. 5A and C). Notably, we did not detect significant changes in type I IFNs (IFN-α and IFN-β, Fig. S5A and B) and IFN-stimulated genes (ISGs) in these mice (Fig. S5C and D).

Previously, we demonstrated that IFN-λ signaling in neutrophils impairs bacterial clearance in adult mice (13). Consistent with this, LysM^Cre^IFNAR1^fl/fl^ and IFNAR1^-/-^ adult mice showed increased IFN-λ secretion (Fig. 2A), but this did not impact lung bacterial burden compared to WT mice (Fig. 1B and C). In contrast, infant mice lacking myeloid cell IFNAR1 exhibited both increased IFN-λ secretion (Fig. 5G) and higher bacterial loads and dissemination (Fig. 4B through E), suggesting age-dependent differences in how type I IFN signaling regulates host defense. Despite enhanced neutrophil recruitment in infant LysM^Cre^IFNAR1^fl/fl^ mice, elevated IFN-λ may impair neutrophil function, contributing to poor bacterial clearance and increased mortality. Together, these data suggest that type I IFN signaling in myeloid cells is a key determinant of disease outcome in infant *B. pertussis* infection.

## DISCUSSION

In this study, we investigated the role of type I IFN signaling in shaping the early immune response to *B. pertussis* infection in both adult and infant mice. We found that type I IFN signaling in myeloid cells contributes to pro-inflammatory cytokine production and lung inflammation. In adult mice, deletion of IFNAR1 specifically in myeloid cells (LysM^Cre^IFNAR1^fl/fl^) significantly reduced lung inflammation without affecting bacterial clearance. Mechanistically, we demonstrated that IFNAR1 signaling inhibits macrophage-derived IFN-λ production. PT induces IFN-λ expression by targeting and inactivating Gi-coupled LPA1 signaling pathways in IFNAR1^-/-^ macrophages, WT neutrophils, and IFNAR1^-/-^ neutrophils. These findings reveal a novel regulatory mechanism by which PT promotes type III IFN responses, particularly in the absence of IFNAR1 signaling, as observed in the lungs of both IFNAR1^-/-^ and LysM^Cre^IFNAR1^fl/fl^ mice. In adult mice, LysM^Cre^IFNAR1^fl/fl^ animals did not show significant differences in lung bacterial burdens compared to controls. However, infant LysM^Cre^IFNAR1^fl/fl^ mice exhibited increased lung bacterial loads and dissemination of bacteria to other organs, as well as increased mortality, indicating that the influence of myeloid-specific type I IFN signaling on host defense is developmentally regulated and more critical during early life. Notably, the increased IFN-λ secretion in these infant mice may impair neutrophil function, despite enhanced neutrophil recruitment, thereby contributing to ineffective bacterial clearance and elevated mortality. Together, our findings highlight a crucial role for type I IFN signaling in myeloid cells in modulating the balance between inflammation and bacterial clearance during *B. pertussis* infection. While this pathway appears dispensable for bacterial control in adult mice, it is essential for protective immunity in infants. These insights provide a foundation for age-targeted strategies aimed at modulating type I IFN signaling to improve outcomes during pertussis infection.

Type I IFNs are induced via extracellular and intracellular pattern recognition receptors (PRRs) of the innate immune system in response to microbial infection (14, 15). The role of type I IFN signaling varies by pathogen and disease context. For instance, *Listeria monocytogenes* induces a robust type I IFN response, which contributes to increased host susceptibility, and mice lacking IFNAR1 are protected from *Listeria* infection (35). In contrast, during *Streptococcus pyogenes* infection, IFNAR1 signaling plays a critical role in restraining excessive pro-inflammatory responses (36). In *Streptococcus pneumoniae* infection, type I IFNs are protective by modulating innate immune cell functions, including those of neutrophils and macrophages (37). In the context of *B. pertussis* infection, we observed a consistent downregulation of pro-inflammatory markers in both adult and infant LysM^Cre^IFNAR1^fl/fl^ mice, indicating that type I IFN signaling in myeloid cells promotes inflammation. However, while adult mice were able to control bacterial burden in the absence of IFNAR1 signaling, infant mice became highly susceptible, displaying increased bacterial loads and dissemination. These findings suggest that although type I IFN signaling drives inflammation, it is also crucial for bacterial clearance during early life. The absence of type I IFN signaling in myeloid cells may dampen inflammatory responses necessary for effective immunity in infants, underscoring an age-dependent requirement for type I IFNs in host defense against *B. pertussis* infection.

Myeloid cells are major effectors of inflammation, and type I IFNs can enhance their activation, survival, and production of pro-inflammatory cytokines, such as TNF-α, IL-6, and IL-1β (38). Loss of IFNAR1 specifically in myeloid cells dampens this inflammatory loop, leading to reduced expression of pro-inflammatory mediators and attenuated lung inflammation. In contrast, whole-body deletion of IFNAR1 eliminates signaling not only in myeloid cells but also in epithelial cells, which rely on type I IFNs for barrier integrity and antimicrobial defense (39). Furthermore, in non-myeloid cells, type I IFNs often play regulatory or anti-inflammatory roles, such as limiting IL-17 responses during bacterial infections and maintaining tissue homeostasis (18). Type I IFNs thus exert context- and cell-type-dependent effects. Our findings that lung inflammation is reduced in LysM^Cre^IFNAR1^fl/fl^ mice but not in whole-body IFNAR1 KO mice suggest that during *B. pertussis infection*, IFNAR1 signaling in myeloid cells promotes inflammation, likely through the amplification of pro-inflammatory cytokine production. In contrast, IFNAR1 signaling in non-myeloid compartments may have protective or regulatory functions. Therefore, whole-body IFNAR1 deficiency may eliminate these beneficial effects and trigger compensatory or dysregulated immune responses that sustain or even exacerbate inflammation.

Our finding that IFN-λ is only produced by IFNAR1 KO macrophages and not by WT macrophages indicates that type I IFN signaling can suppress IFN-λ induction, possibly in a cell-specific manner, since IFN-λ was produced by WT neutrophils. This may be due to type I IFN induction of ISGs, many of which act as transcriptional repressors or negative regulators of pattern recognition receptor (PRR) signaling (40). Among these, suppressor of cytokine signaling (SOCS) proteins inhibit JAK-STAT signaling, a pathway critical for IFN-λ production (41), while ubiquitin-specific protease 18 (USP18) has also been shown to dampen IFN-λ expression (42). Thus, type I IFN signaling in macrophages may suppress IFN-λ expression through a combination of feedback inhibition (via SOCS and USP18), competition for transcriptional coactivators, and the predominance of STAT1/2 signaling pathways, which may interfere with IRF3/7-mediated induction of IFN-λ gene expression (43). This suppression likely serves to inhibit IFN-λ responses in macrophages.

Lysophosphatidic acid receptor 1 (LPA1) is expressed on myeloid cells (30) and functions as a Gi-coupled G protein-coupled receptor (GPCR) (29), which is a known target of PT. LPA1 plays a critical role in the regulation of immune responses during infection, notably by suppressing the production of type I and III interferons, thereby impairing host antiviral defense (31). We have previously shown that both murine and human neutrophils secrete IFN-λ in a PT-dependent manner and that this IFN-λ intrinsically inhibits neutrophil antimicrobial function (13). In this study, we report that both neutrophils and IFNAR1^-/-^ macrophages secrete IFN-λ in a manner dependent on the Gi-coupled receptor LPA1. Consistent with this, we observed elevated levels of IFN-λ in the lungs of both adult and infant LysM^Cre^IFNAR1^fl/fl^ mice. Notably, while increased IFN-λ production in adult mice did not significantly impact bacterial burden, in infant mice, it was associated with heightened susceptibility to *B. pertussis* infection, including increased bacterial loads and systemic dissemination. These findings suggest that although IFN-λ may play a protective or neutral role in adults, its overproduction in infants lacking type I IFN signaling in myeloid cells impairs neutrophil function, contributing to bacterial persistence and increased lethality. This highlights an age-dependent divergence in the role of IFN-λ during pertussis infection, underscoring the importance of tightly regulated IFN signaling for effective immune defense in early life.

The cause of death in these infant mice is likely due to the activity of PT, since we have previously shown that PT is the major *B. pertussis* virulence factor responsible for mortality in P7 infant mice via induction of leukocytosis and pulmonary hypertension (1, 2). In the current study, we observed increased expression of IFN-λ in the lungs of myeloid cell-specific IFNAR1-deficient infant mice following *B. pertussis* infection. Our earlier work demonstrated that PT-induced IFN-λ impairs neutrophil function, leading to reduced bacterial clearance and exacerbated lung inflammation (13). These findings suggest that the PT-dependent elevated IFN-λ levels observed in IFNAR1-deficient infant mice may contribute to impaired bacterial clearance, resulting in increased bacterial burden and dissemination. This effect appears to persist even through P21, indicating a sustained impact of IFN-λ–mediated immune dysregulation in early life and lethality.

Neutrophils are essential components of the innate immune system, serving as the first line of defense against bacterial infections. However, dysregulated neutrophil recruitment can result in excessive inflammation and tissue damage, particularly in infants with immature immune systems (44). In our study, infected LysM^Cre^IFNAR1^fl/fl^ P10 infant mice exhibited a higher influx of neutrophils and increased pulmonary inflammation compared to WT P10 mice, despite displaying reduced expression of pro-inflammatory markers and elevated levels of the anti-inflammatory cytokine IL-10. Notably, *Bordetella parapertussis* has been shown to induce lung IL-10 production in mice, facilitating immune evasion and bacterial persistence (45), suggesting a role for IL-10 in dampening protective host responses. These findings are further supported by our observation that myeloid-specific deletion of type I IFN signaling in infant mice increased lung IL-10 expression, highlighting the protective role of type I IFN signaling in this age group. We have previously shown that constitutive type I IFN signaling in infant IFNAR1-SA mice enhances survival following *B. pertussis* infection, which is associated with elevated pro-inflammatory cytokine expression and significant suppression of IL-10 (5). In contrast, in myeloid cell-specific IFNAR1 KO infant mice, we observed a marked reduction in pro-inflammatory cytokines and a concomitant increase in IL-10 levels. This shift likely creates an immunosuppressive environment, impairing effective bacterial clearance and promoting increased bacterial load and systemic dissemination, ultimately contributing to the observed lethality in these mice.

Collectively, our data add to the growing body of evidence that the immune response to bacterial pathogens is developmentally regulated and that type I IFN signaling plays a distinct, age-dependent role in modulating disease outcomes during *B. pertussis* infection. While the therapeutic potential of modulating type I IFNs in pertussis remains to be fully explored, it is noteworthy that IFN-α has been approved by the FDA for use in cancer immunotherapy (46). However, based on our findings, targeted induction of type I IFN signaling on myeloid cells may offer a more effective therapeutic strategy for treating pertussis in highly susceptible infant populations.

## SUPPLEMENTAL MATERIAL

Supplemental material iai.00306-25-s0001.docx Fig. S1 to S5; Table S1.

ASM does not own the copyrights to Supplemental Material that may be linked to, or accessed through, an article. The authors have granted ASM a non-exclusive, world-wide license to publish the Supplemental Material files. Please contact the corresponding author directly for reuse.
