# Supplementary material for: Myeloid cell-specific type I interferon signaling mediates age-dependent inflammation and protection in Bordetella pertussis infection
Source: Infect Immun. 2025 Sep 22;93(10):e00306-25. doi: 10.1128/iai.00306-25 (PMC12519780; doi:10.1128/iai.00306-25)
Supplement: Supplemental material — Fig. S1 to S5; Table S1. [file iai.00306-25-s0001.docx]

**
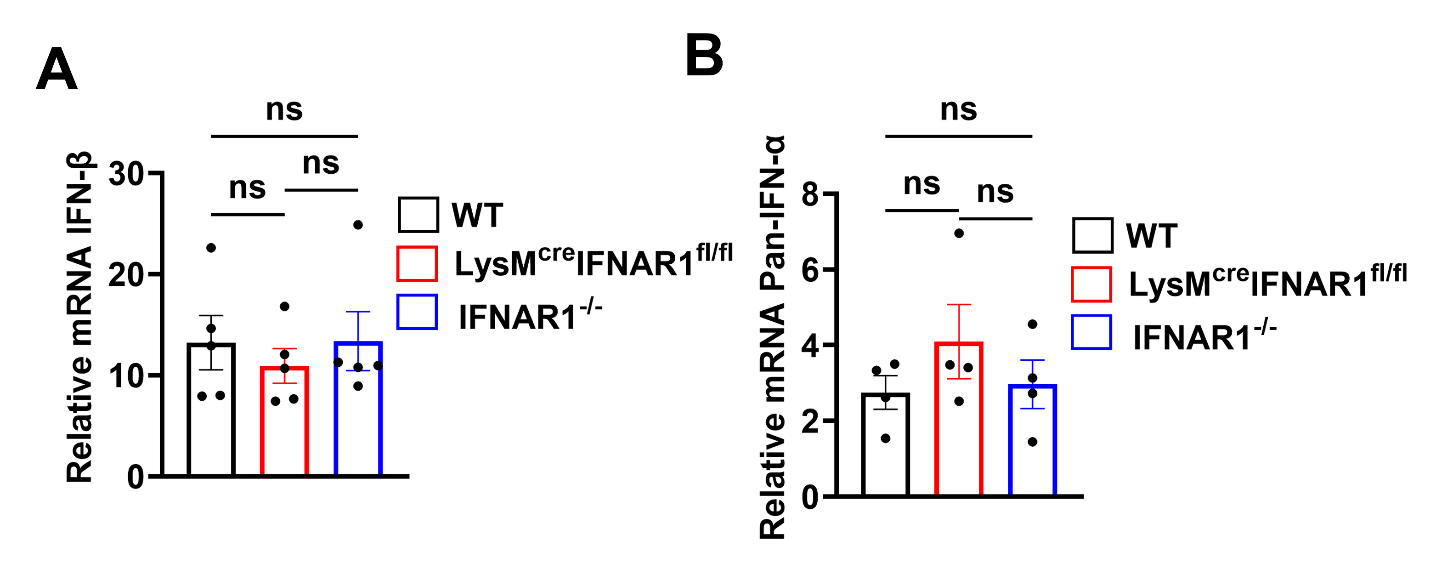
**

**Supplementary Figure 1.**  Adult mice (n>4) were euthanized on day 7 post-inoculation with *B. pertussis* or PBS sham inoculum, and lungs were dissected for assessment of mRNA levels of (A) IFN-β and (B) Pan-IFN-α by quantitative RT-PCR (infected versus sham-inoculated). ns not significant, by one-way ANOVA.


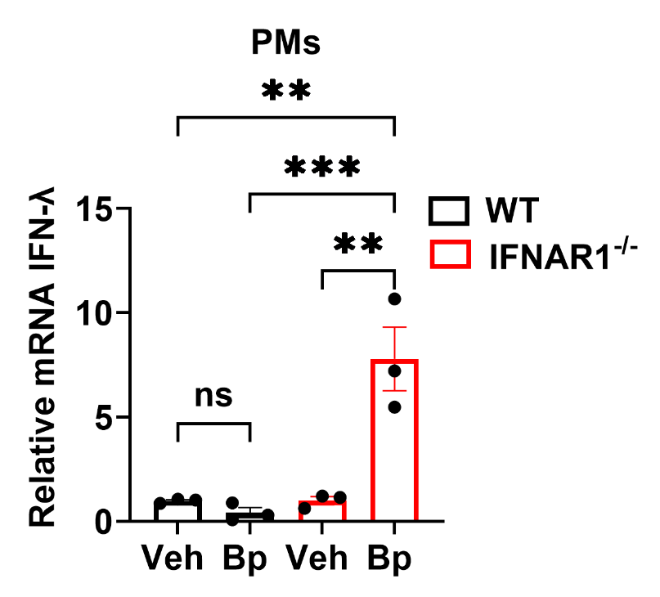


**Supplementary Figure 2.** Type III IFN responses of wild-type (WT) and IFNAR1^-/-^ peritoneal macrophages (PMs) following stimulation with *B. pertussis* (Bp, MOI 10). Samples were collected at 8 h post-stimulation for assessment of IFN-λ mRNA by quantitative RT-PCR (infected versus sham-inoculated). **p < 0.01, ***p < 0.001, ns not significant, by one-way ANOVA.


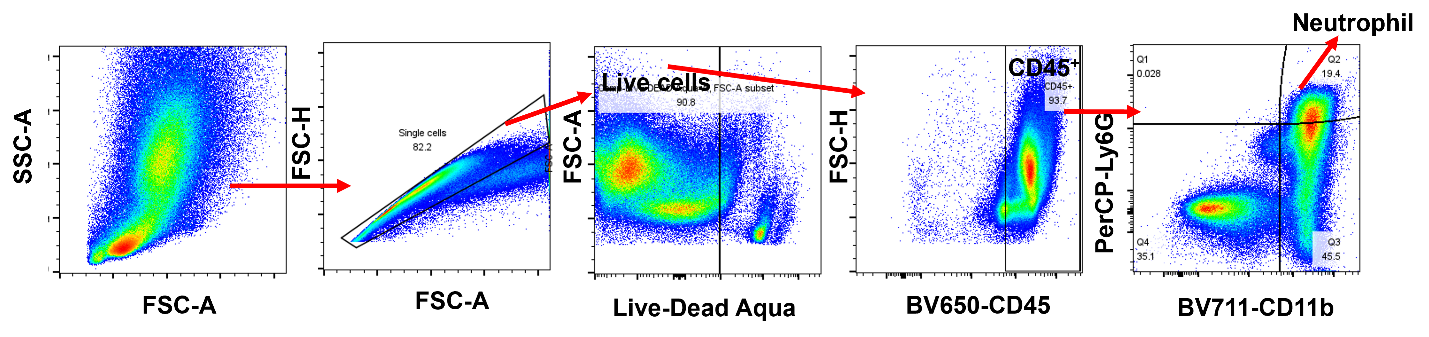


**Supplementary Figure 3.** Gating strategy used to identify the neutrophil population in P10 infected mouse lungs. Single-cell suspensions were prepared from enzymatically digested mouse lungs. After exclusion of doublets and debris, immune cells were identified based on CD45 expression. Neutrophils were gated as CD45⁺Ly6G⁺CD11b⁺ cells.


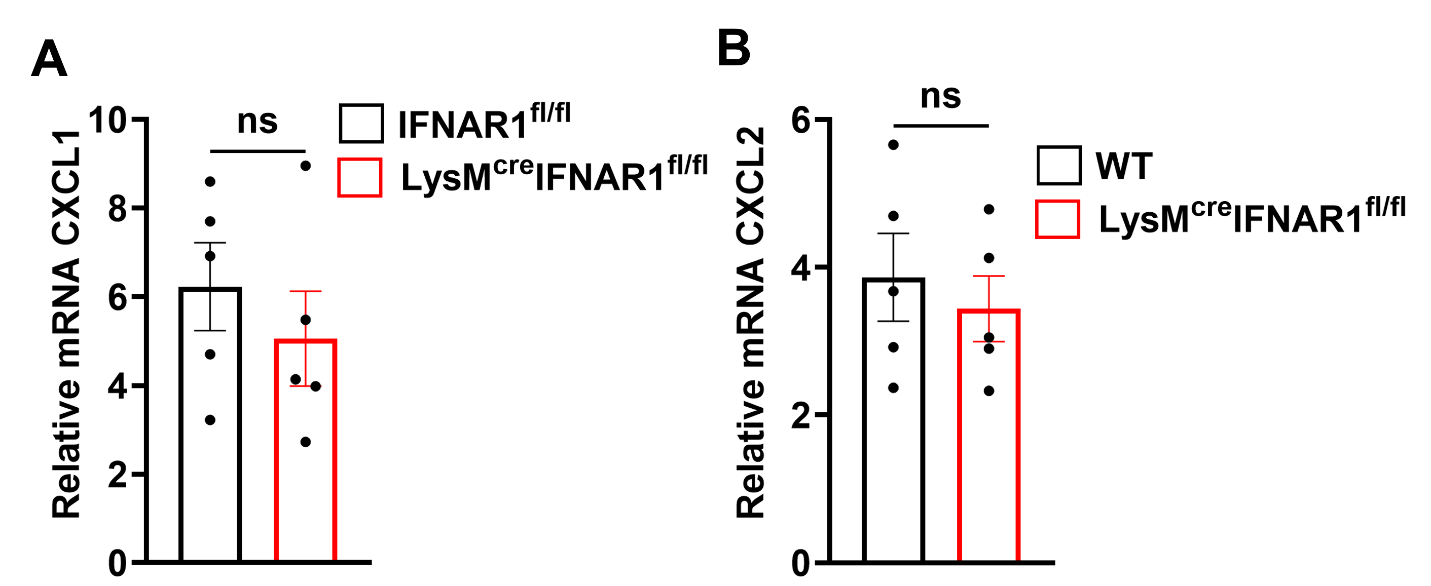


**Supplementary Figure 4.** P10 infant mice (n>4) were euthanized on day 7 post-inoculation with *B. pertussis* or PBS sham inoculum, and lungs were dissected for assessment of mRNA levels of (A) CXCL1 and (B) CXCL2 by quantitative RT-PCR (infected versus sham inoculated). ns not significant, by one-way ANOVA.


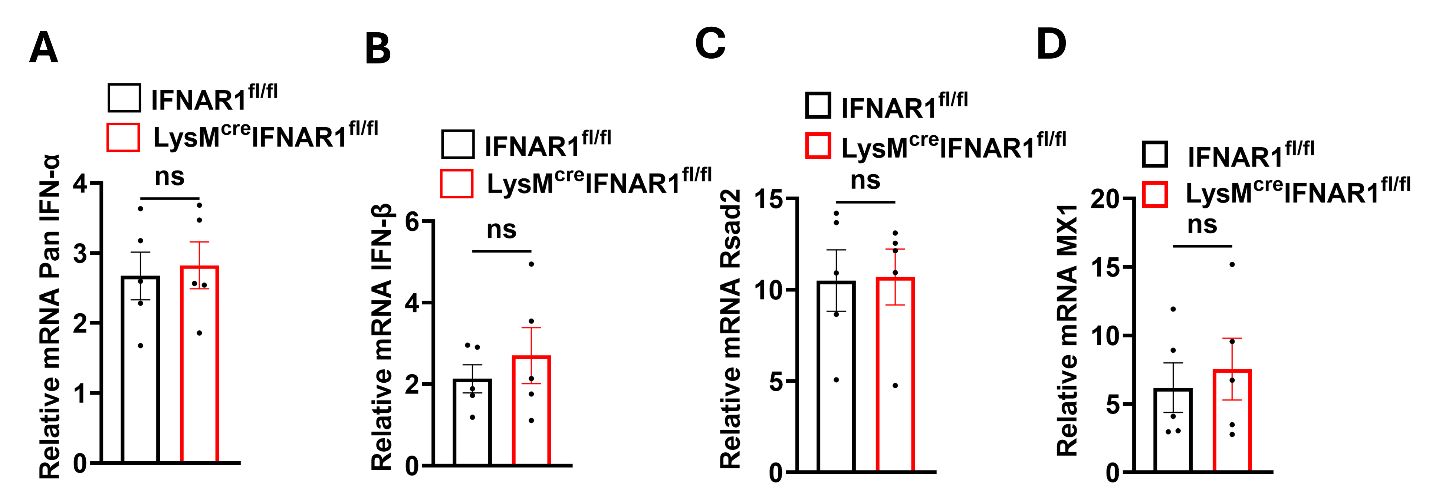
**Supplementary Figure 5.** P10 infant mice (n>4) were euthanized on day 7 post-inoculation with *B. pertussis* or PBS sham inoculum, and lungs were dissected for assessment of mRNA levels of (A) Pan IFN-α, (B) IFN-β, (C) Rsad2, and (D) MX1 by quantitative RT-PCR (infected versus sham inoculated). ns not significant by, one-way ANOVA.

**Supplemental Table I:** Primer sequences for qRT-PCR analysis of mouse gene expression**.**

| Gene | Forward Primer | Reverse Primer |
| --- | --- | --- |
| HPRT | 5′-GCTGACCTGCTGGATTACATTAA-3’ | 5′- GATCATTACAGTAGCTCTTCAGTCTG-3′ |
| Pan-IFNα | 5′-CCAGCAGGGCGTCTTCCT-3’ | 5′-GCAACCCTCCTAGACTCATTCT-3’ |
| IFNβ | 5′-CCCTATGGAGATGACGGAGA-3’ | 5′-CTGTCTGCTGGTGGAGTTCA-3’ |
| IFN-λ2/3 | 5′-GACAAGAACCCAAGCTGACC-3’ | 5′-ACCTCAGGTCCTTCTCAAGC-3’ |
| Rsad2 | 5′-TGCTATCTCCTGCGACAGCTT-3’ | 5′-CCTTGACCACGGCCAATC-3’ |
| MX1 | 5′-TGGACATTGCTACCACAGAGGC-3’ | 5′-TTGCCTTCAGCACCTCTGTCCA-3’ |
| CXCL1 | 5′-TCCAGAGCTTGAAGGTGTTGCC-3’ | 5′-AACCAAGGGAGCTTCAGGGTCA-3’ |
| CXCL2 | 5′-CATCCAGAGCTTGAGTGTGACG-3’ | 5′-GGCTTCAGGGTCAAGGCAAACT-3’ |
